# Human Norovirus Induces Aquaporin 1 Production by Activating NF-κB Signaling Pathway

**DOI:** 10.3390/v14040842

**Published:** 2022-04-18

**Authors:** Mudan Zhang, Binman Zhang, Rui Chen, Miaomiao Li, Zifeng Zheng, Wanfu Xu, Yifan Zhang, Sitang Gong, Qinxue Hu

**Affiliations:** 1 Department of Gastroenterology, Guangzhou Women and Children’s Medical Center, Guangzhou Medical University, Guangzhou 510623, China; mudan@wh.iov.cn (M.Z.); zzf870806@163.com (Z.Z.); xushi123@gmail.com (W.X.); 2 State Key Laboratory of Virology, Wuhan Institute of Virology, Center for Biosafety Mega-Science, Chinese Academy of Sciences, Wuhan 430071, China; zbm199501@sina.com (B.Z.); chenrui880110@163.com (R.C.); 117723892008@163.com (M.L.); 3 University of Chinese Academy of Sciences, Beijing 100049, China; 4 Maternal and Child Hospital of Hubei Province, Tongji Medical College, Huazhong University of Science and Technology, Wuhan 430070, China; zyf197538@163.com; 5 Institute for Infection and Immunity, St George’s, University of London, London SW17 0RE, UK

**Keywords:** human norovirus, aquaporin 1, NF-κB, p65

## Abstract

Human norovirus (HuNoV) is one of the major pathogens of acute nonbacterial gastroenteritis. Due to the lack of a robust and reproducible in vitro culture system and an appropriate animal model, the mechanism underlying HuNoV-caused diarrhea remains unknown. In the current study, we found that HuNoV transfection induced the expression of aquaporin 1 (AQP1), which was further confirmed in the context of virus infection, whereas the enterovirus EV71 (enterovirus 71) did not have such an effect. We further revealed that VP1, the major capsid protein of HuNoV, was crucial in promoting AQP1 expression. Mechanistically, HuNoV induces AQP1 production through the NF-κB signaling pathway via inducing the expression, phosphorylation and nuclear translocation of p65. By using a model of human intestinal epithelial barrier (IEB), we demonstrated that HuNoV and VP1-mediated enhancement of small molecule permeability is associated with the AQP1 channel. Collectively, we revealed that HuNoV induced the production of AQP1 by activating the NF-κB signaling pathway. The findings in this study provide a basis for further understanding the significance of HuNoV-induced AQP1 expression and the potential mechanism underlying HuNoV-caused diarrhea.

## 1. Introduction

Noroviruses (NoVs) are non-enveloped, linear, single-stranded, positive sense RNA viruses belonging to genus Norovirus of the family Caliciviridae [1,2]. NoVs are divided into ten genogroups (GI-GX) that are subdivided into more than 49 genotypes [3], of which GI, GII and GIV cause illness in humans [4]. Human norovirus (HuNoV) is one major cause of acute gastroenteritis worldwide [5]. The virus spreads by fecal–oral route and is highly contagious with as few as 18 virus particles being able to establish infection [6]. Currently, most gastroenteritis outbreaks are caused by GII.4 genotype, although cases caused by other genotype such as GII.17 are rising [7,8,9].

HuNoV often breaks out in semi-closed communities such as schools, sanatoriums, hospitals, cruise ships and disaster relief agencies [10,11,12]. All age groups, especially infants, the elderly and immunocompromised patients, are susceptible to HuNoV. Globally, norovirus causes an estimated 699 million illnesses and 219,000 deaths each year, resulting in >$4 billion in direct medical costs and >$60 billion in indirect medical costs [13,14,15]. Although HuNoV causes a significant worldwide disease burden due to health care costs and loss of productivity, it is still unknown concerning the potential mechanism of HuNoV-induced diarrhea, which is largely due to the lack of a robust and reproducible in vitro culture system and a good animal model. Although it has been reported that human intestinal enteroids (HIEs) can support HuNoV infection in vitro [16,17,18], the scarcity of clinically discarded human intestinal tissues and the complex construction process of HIEs limit its application.

One typical symptom of HuNoV infection is diarrhea, which is the second leading cause of death in children under 5 years old in the world [5,19]. It is known that one direct cause of diarrhea is the abnormal expression of aquaporin (AQP) (regardless of upregulation or downregulation) in the intestinal tissue [20,21,22,23]. AQP is located on the cell membrane and divided into at least 13 AQP (AQP0–AQP12) expressed in different tissues [24]. AQP1, AQP2, AQP3, AQP4 and AQP8 expressed in the colon were reported to mainly be composed of “pores” on the cell membrane, playing a key role in regulating the dynamic balance of water [25,26]. Previous studies showed that the expression of AQP1, AQP3, AQP7 and AQP8 in patients with inflammatory bowel disease was significantly decreased [27], whereas the expression of AQP3 was significantly increased in the process of magnesium phosphate-induced diarrhea [22].

In this study, we found that HuNoV cDNA clone induced AQP1 expression upon transfection in the human intestinal epithelial cell line Caco2. In the context of virus infection, HuNoV but not the enterovirus EV71 induced AQP1 expression. We further revealed that the major capsid protein VP1 is crucial for promoting AQP1 expression. Mechanistically, HuNoV induces AQP1 production through the NF-κB signaling pathway via inducing the expression, phosphorylation and nuclear translocation of p65. In addition, we demonstrated that HuNoV and VP1 increased the permeability of the intestinal epithelial barrier, which is associated with the AQP1 channel. Our findings highlight the importance of HuNoV-induced AQP1 expression, which may shed light on the mechanism underlying HuNoV-caused diarrhea.

## 2. Materials and Methods

### 2.1. Viruses, Cell Lines, Antibodies and Inhibitors

HuNoV (GII.4 strain)-positive stool samples were obtained from diarrhea patients in the Guangzhou Women and Children’s Medical Center. The sequence of our clinical isolate HuNoV GII.4 has been uploaded to GenBank and assigned to number OL721917. The full-length HuNoV cDNA clone constructed in the Wuhan Institute of Virology (unpublished data) was transfected into HEK293T cells to produce HuNoV progeny viruses. The recombinant lentivirus expressing our isolated HuNoV complete cDNA or its VP1 constructed in our laboratory (unpublished data), together with the helper plasmids psPAX-2 and pMD-2G, were co-transfected into HEK293T cells to produce lentivirus carrying HuNoV (named lenti-HuNoV) or VP1 cDNA (named lenti-VP1). The lenti-HuNoV or VP1 was titrated by counting the number of GFP-positive cells after serial dilution. EV71 was kindly provided by Dr. Zhenhua Zheng (Wuhan Institute of Virology, Chinese Academy of Sciences, Wuhan, China). Virus stocks were aliquoted and stored at −80 °C before being used for infection. HEK293T cells and human colon epithelial cell line Caco2 were purchased from ATCC and cultured in Dulbecco’s modified Eagle medium (DMEM) (ThermoFisher, 12430062, Scoresby, Australia) supplemented with 10% FBS, 100 units/mL penicillin and streptomycin each at 37 °C in a 5% CO_2_ incubator.

Abs against AQP1, AQP4, p65, PCNA and β-actin were purchased from Proteintech Biotechnology (20333-1-AP, 16473-1-AP, 10745-1-AP, 10205-2-AP and 60008-1-Ig, Wuhan, China). Ab against phospho-p65 was purchased from Cell Signaling Technology (3033S). Mouse sera against HuNoV capsid protein VP1 were produced by immunizing mice with eukaryotically expressed VP1 protein in our laboratory. VP1 specific siRNA was synthesized by Guangzhou Ruibo Biotechnology (Guangzhou, China). A human p65 shRNA plasmid kit was purchased from OriGene (TR302038, Beijing, China). The inhibitor bacopaside II against AQP1 channel was purchased from MCE (HY-N6016, Wuhan, China).

### 2.2. Plasmid Construction

The HuNoV genome was extracted from a HuNoV-positive stool sample using a QIAamp RNA Blood Mini Kit (Qiagen, 52304, Hilden, Germany). HuNoV cDNA was synthesized with moloney murine leukemia virus transcriptase (Promega, M170B, Dane County, WI, USA). The newly synthesized cDNA was used as the template to amplify full-length HuNoV cDNA and VP1. The open-reading frame (ORF) encoding VP1 was amplified by PCR with the primers shown in Supplementary Materials Table S1. A C-terminal 3 × Flag tag was introduced into VP1 by the reverse primer. The PCR product was cloned into pcDNA3.1(+) (Invitrogen, Waltham, MA, USA) and the constructed expression plasmid was named VP1-C3F. The promoter reporters AQP1-Luc and AQP4-Luc were constructed using pGL3-basic as the backbone plasmid. The primers of AQP1 and AQP4 were shown in Supplementary Materials Table S1. All constructs were verified by DNA sequencing (Sunny Biotechnology, Shanghai, China).

### 2.3. Luciferase Reporter Assay

Caco2 cells were seeded in 24-well plates overnight and co-transfected with empty vector or plasmid encoding full-length HuNoV cDNA or VP1 and reporter plasmid AQP1-Luc or AQP4-Luc. In some cases, Caco2 cells were co-transfected with empty vector or plasmid encoding full-length HuNoV cDNA or VP1 and IRF-3-responsive reporter plasmid PRD(III-I)_4_-Luc or NF-κB-responsive reporter plasmid NF-κB-Luc, or co-transfected with empty vector and PRD(III-I)_4_-Luc or NF-κB-Luc followed by stimulation with SeV (Sendai virus) at 4 h post-transfection. Transfections were carried out using X-tremeGENE™ HP DNA Transfection Reagent (Roche, 6366236001, Penzberg, Germany) according to the manufacturer’s instructions. At 24 h post-transfection, cells were lysed to measure the activity of Firefly luciferase (Promega, E1501, Madison, WI, USA) according to the manufacturer’s instructions. Values for the samples were expressed as fold increase in the value in cells transfected with empty vector.

### 2.4. RNA Isolation and Quantitative PCR

Cells were harvested to extract total RNA using the RNA isolation kit (Macherey-Nagel, 740955, Dueren, Germany) according to the manufacturer’s instructions. The cDNA was synthesized with Moloney murine leukemia virus transcriptase (Promega, M170B, Madison, WI, USA). The newly synthesized cDNA was used as the template to amplify the genes of AQP1, AQP4 and GAPDH. The primer pairs for AQP1 and AQP4 were named AQP1-F/AQP1-R and AQP4-F/AQP4-R (Supplementary Materials Table S1), respectively. GAPDH was used as an internal control and amplified with primers GAPDH-F and GAPDH-R (Supplementary Materials Table S1). Relative real-time quantitative PCR was also performed on an ABI StepOne apparatus using a SYBR Green Real-Time PCR Master Mix (Toyobo, QPK-201, Osaka, Japan) according to the following conditions: 95 °C for 1 min, followed by 40 cycles of 95 °C for 15 s, 60 °C for 15 s, and 72 °C for 45 s. The expression difference was calculated on the basis of 2^−ΔΔ*C*t^ values. In some cases, the copies of HuNoV genome were quantified and performed on ABI StepOne apparatus using Norovirus GII nucleic acid detection kit (DAAN GENE, DA2811, Guangzhou, China) according to the manufacturer’s instructions.

### 2.5. Immunofluorescence Assay (IFA)

Caco2 cells were seeded in 35 mm dishes with glass bottoms overnight and transfected with plasmid expressing full-length HuNoV cDNA or VP1. At 24 h post-transfection, cells were fixed with 4% formaldehyde and permeabilized with 0.2% Triton X-100. After three washes with 1 × PBS, cells were blocked with 5% BSA at 4 °C overnight. Thereafter, cells were incubated with rabbit anti-p65 Ab at a dilution of 1:200 at 37 °C for 1 h. Following three washes with 1 × PBS, cells were then incubated with Alexa Fluor 647-conjugated goat anti-rabbit IgG (Invitrogen, A-21244, Waltham, MA, USA) at a dilution of 1:1000 for 1 h at 37 °C. Cells were subsequently washed and incubated with DAPI for 10 min at 37 °C. After extensive washes, cells were observed under a fluorescence microscope (PerkinElmer UltraVIEW VoX, Waltham, MA, USA).

### 2.6. Western Blot (WB)

WB analysis was performed as described previously [28,29]. Briefly, cells were lysed with WB/IP lysis buffer (Beyotime Biotechnology, P0013, Shanghai, China) containing protease inhibitor cocktail (Roche, 11697498001, Germany). Cell extracts were subjected to 10% SDS-PAGE, transferred onto 0.45 μm PVDF membranes (Millipore, Darmstadt, Germany), and blocked with 5% non-fat milk at 4 °C overnight. The membrane was clipped and probed with a primary antibody at room temperature for 2 h. After washes with TBST, the membrane was incubated with horseradish peroxidase (HRP)-conjugated goat anti-rabbit IgG (BOSTER, BA1054, Wuhan, China) or goat anti-mouse IgG (BOSTER, BA1051, Wuhan, China) at room temperature for 1 h. Protein bands were exposed in an Image Lab^TM^ System (BIO-RAD, Contra Costa County, CA, USA) after the addition of chemiluminescent substrate (Advansta, K-12045-D50, San Jose, CA, USA). The relative intensities of Western blots were quantified using Image J. A protein molecular weight marker was purchased from Bioscience (Double Helix, P12083, Shanghai, China).

### 2.7. HIE (Human Intestinal Enteroids) Construction

#### 2.7.1. Human Crypts Isolation

Discarded human intestinal tissues were collected from the Department of Gastroenterology, Guangzhou Women and Children’s Medical Center. The isolation of human crypts from human intestinal tissues was conducted as described previously [16,30,31]. Briefly, tissues were washed and cut to remove fat as much as possible. For HIE construction, tissues were cut into about 5 mm pieces and washed with precooled PBS several times. After immersed in cold chelation solution (CCS) (5.6 mmol/L Na2HPO4, 8 mmol/L KH2PO4, 96.2 mmol/L NaCl, 1.6 mmol/L KCl, 43.4 mmol/L sucrose, 54.9 mmol/L D-sorbitol, 0.5 mmol/L DL-dithiothreitol and 2 mmol/L EDTA) at 4 °C overnight, the tissues were mechanically pipetted up and down several times and bathed at 4 °C for 1 min. The supernatant was collected and observed to check whether the intestinal crypts were isolated from tissues. The remaining tissues were repeatedly blown to promote the isolation of crypts from tissues using new CCS until crypts were not observed under microscope. The collected crypts in CCS were centrifuged at 200× *g* for 3 min and then resuspended with 30 mL TrypLE Expression (GIBCO, 12604-021, Waltham, MA, USA) to digest at 37 °C for 1 h. Subsequently, the digested crypts were dispersed and centrifuged at 1200 rpm for 3 min. DMEM/F12 medium (GIBCO, SH30023.01, USA) supplemented with 10% FBS, 100 units/mL penicillin and streptomycin each (Complete DMEM/F12) were added to resuspend the separated cells. If the cells were not in a single dispersed state, 0.25% trypsin were added to further digest the cells at 37 °C for 3 min. The cells were then filtered through 40 μm cell strainer (BD Biosciences, 352340, Franklin Lakes, NJ, USA) and washed with PBS. After centrifugation at 1200 rpm for 5 min, cells were resuspended and counted to construct three-dimensional (3D) culture of HIEs.

#### 2.7.2. Three-Dimensional (3D) Culture of HIEs

Matrigel (CORNING, 354230, Corning, NY, USA) was placed at 4 °C overnight, and pipette tips, injectors and collection tubes were all pre-chilled in an ice bath. 10^4^ cells were resuspended with 25 μL Matrigel, and 40 μL cell suspension was added into one well of 24-well plates, followed by solidification at 37 °C in a 5% CO_2_ incubator for 10 min. Thereafter, 500 μL 37 °C complete DMEM/F12 medium was added into the well, which was supplemented with 50% L-WNT3A, 20% R-spondin 1, 10% Noggin-Fc, 1 × B27 (Invitrogen, 17504-044, USA), 1 × N2 (Invitrogen, 15502-048, USA), 1 mM N-acetylcysteine (Sigma-Aldrich, A0737, St. Louis, MO, USA), 50 ng/mL EGF (Invitrogen, PMG8044, USA), 10 mM nicotinamide (Sigma-Aldrich, N0636, USA), 500 nM A-83-01 (Tocris, 2939, Shanghai, China), 10 μM SB202190 (Sigma-Aldrich, S7067, USA), 10 μM Y-27632 (MedChemExpress, HY-10071, China) and 10 nM Gastrin I (Sigma-Aldrich, 05-23-2301, USA). The above complete medium with growth factors was named CMGF+. Culture was refreshed using CMGF+ medium every other day until the HIEs were ready to be passaged.

#### 2.7.3. HIE Passage and Differentiation

The old medium was removed from wells, leaving the Matrigel plug intact. Then, 500 μL cold CMGF+ was added into the wells, which was followed by mechanically pipetting up and down to break up Matrigel. The suspension was collected after being pipetted up and down several times using a 1 mL cold syringe with a 25G 5/800 needle. Subsequently, two volumes of cold DMEM/F12 medium were added to further dissolve the Matrigel. Cells were centrifuged at 1200 rpm for 5 min and then plated with Matrigel in 24-well plates. In some cases, complete DMEM/F12 was switched to differentiation medium. Culture was refreshed with differentiation medium every other day before the HIE was ready for experiments. The differentiation medium contained the same components as those of CMGF+ medium without the addition of L-WNT3A, R-Spondin1, SB202190, nicotinamide and Y-27632, and had 50% reduction of Noggin-Fc.

After differentiation for 7 days, HIEs were released from the 3D-culture model and seeded in 6-well plates overnight, which was followed by infection with HuNoV at a genome copy of 3.5 × 10^7^. At 12, 24, 48 and 72 h post-infection, HIEs were harvested to extract total RNA using RNA isolation kit (Macherey-Nagel, 740955, Dueren, Germany). The copies of the HuNoV genome were evaluated by real-time quantitative PCR using Norovirus GII nucleic acid detection kit (DAAN GENE, DA2811, Guangzhou, China).

### 2.8. Human IEB (Intestinal Epithelial Barrier) Construction and Application

#### 2.8.1. IEB Construction

Caco2 cells were propagated and maintained at 37 °C in a 5% CO_2_ incubator. 3 × 10^4^ cells were seeded into permeable polyester membrane filter supports (Millipore, MCHT24H48, Germany), which were pre-coated with 30 μg collagen IV (BIOSS, bs-0806P, Beijing, China). The apical and basolateral volume of culture medium were 200 μL and 900 μL, respectively. Caco2 cells were used between passage 40 and 55, and the medium was refreshed every other day until use. The IEB was apically infected with lentivirus-transduced HuNoV (lenti-HuNoV) or VP1 (lenti-VP1) at a multiplicity of infection 1 (MOI = 1) for 3 d when the resistance value was around 500 Ω·cm^2^ at day 14 post-cultivation.

#### 2.8.2. Trans-Epithelial Electrical Resistance (TEER)

TEER was measured using a voltmeter (Millipore, Millicell ERS-2, Middlesex County, MA, USA) at 3 d post-infection according to the manufacturer’s protocol. After subtraction of the TEER values in the blank bathing solution, the values of mock infection were normalized and represented as 100%. Values for the samples were expressed as percentages of the value mock-infected with DMEM.

#### 2.8.3. Paracellular Permeability

IEB was pretreated with or without the inhibitor of AQP1 channel, 10 μM bacopaside II, for 1 h, which was followed by the addition of 4 kDa FITC-conjugated dextran (1 mg/mL) to the upper chamber of Transwells. After 2 h, 4 h or 6 h incubation, 100 μL of sample was collected from the lower chamber to quantify the amount of FITC–dextran penetrated from the upper to lower chamber. Meanwhile, 100 μL DMEM was supplemented into the lower chamber. Fluorescence was read using a VARIOSKAN FLASH instrument (Thermo Scientific, Waltham, MA, USA) that detects the fluorescence at excitation and emission wavelengths of 495 nm and 519 nm, respectively. The values of the samples were expressed as fold increase in the fluorescence intensity in mock-infection.

### 2.9. Negative Staining for Transmission Electron Microscopy

A carbon-supported membrane with a 230 mesh size was inserted into the purified HuNoV suspension. After 8 min absorption, the membrane was then softly transferred into 2% phosphotungstic acid solution for 10 min. After several washes with ultrapure water, the membrane was taken out and naturally dried at least for 12 h. Then, the sample absorbed onto the membrane was observed under Transmission Electron Microscopy (100 kv, HITACHI, Tokyo, Japan).

### 2.10. Statistical Analysis

All experiments were repeated at least three times, and the data are presented as mean ± S.D. with each condition performed in triplicate or in duplicate unless otherwise specified. Data analyses were performed with GraphPad Prism 8 software (GraphPad, San Diego, CA, USA). Comparison between two groups was analyzed by a two-tailed unpaired Student’s t-test, whereas comparisons among more than two groups were analyzed by one-way ANOVA with the Tukey’s test. The relative intensities of Western blots were quantified using Image J. *p* < 0.05 was considered statistically significant.

## 3. Results

### 3.1. HuNoV Transfection Promotes AQP1 Expression

**Figure 1:**
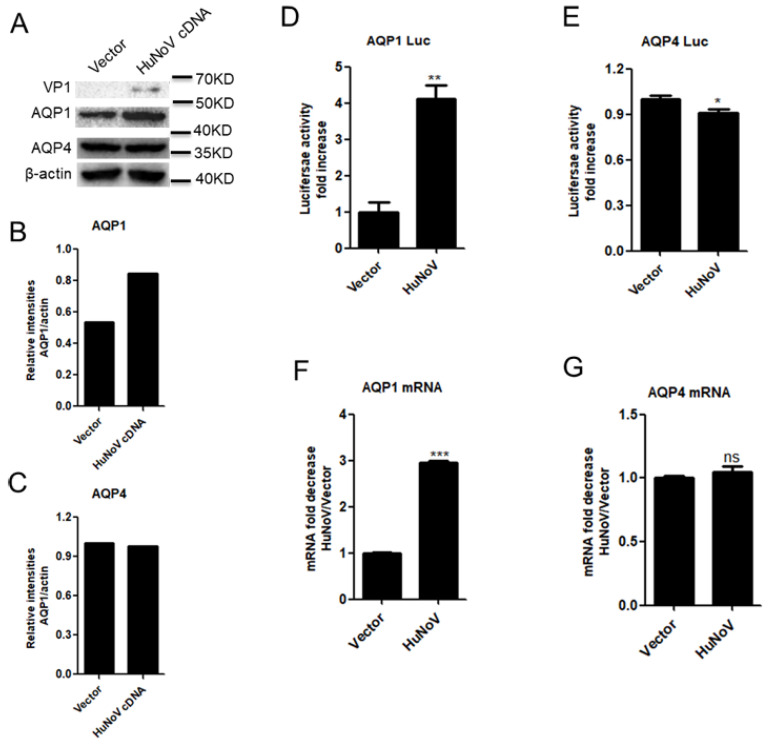
HuNoV transfection promotes AQP1 expression. (**A**–**C**). HuNoV transfection induced the expression of AQP1 in Caco2 cells. Caco2 cells were transfected with full-length HuNoV cDNA-expressing plasmid or empty vector for 24 h. The protein levels of AQP1, AQP4 and HuNoV VP1 were detected by WB (**A**) and quantified using Image J (**B**,**C**). (**D**,**E**). HuNoV promoted the activation of AQP1 promoter. Caco2 cells were co-transfected with empty vector or full-length HuNoV cDNA-expressing plasmid and AQP1-Luc or AQP4-Luc for 24 h. Cells were lysed and detected for the activity of Firefly luciferase. Values for the samples were expressed as a fold increase in the value induced in empty vector-transfected samples. (**F**,**G**). HuNoV promoted the transcription of AQP1. Caco2 cells were transfected with empty vector or full-length HuNoV cDNA-expressing plasmid for 24 h. Cells were harvested, and total RNA was extracted. The expression of AQP1, AQP4 and GAPDH genes was evaluated by relative real-time quantitative PCR. AQP1 and AQP4 mRNA copies were normalized using GAPDH and expressed as fold increase in the value for the empty vector (**F**,**G**). Data shown are mean ± S.D. of three independent experiments with each condition performed in triplicate. One representative experiment out of three is shown. * *p* < 0.05, ** *p* < 0.01, *** *p* < 0.001, ns, not significantly.

Diarrhea is one typical symptom of HuNoV infection. Given that the abnormal expression of AQPs contributes to diarrhea [32,33,34,35,36,37], we asked whether HuNoV-caused diarrhea is associated with AQP abnormal expression. We first constructed a full-length HuNoV cDNA clone. Following transfection into Caco2 cells, the full-length HuNoV cDNA clone produced progeny viruses containing intact RNA genome with the size similar to that of HuNoV, although the progeny viruses were unable to infect Caco2 cells (Supplementary Materials Figure S1A–E). Considering that AQP1 and AQP4 are highly expressed in the colon, we first test whether HuNoV regulates the expression of AQP1 and AQP4 in human colon epithelial cells Caco2 using the full-length HuNoV cDNA clone. Caco2 cells were transfected with the full-length HuNoV cDNA for 24 h. The results showed that HuNoV transfection promoted the production of AQP1 but not AQP4 in Caco2 cells, while the expression of viral protein VP1 was confirmed after transfection with complete HuNoV cDNA (Figure 1A–C). Likewise, HuNoV transfection only promoted the activation of AQP1 promoter (Figure 1D) and the transcription of AQP1 (Figure 1F) but not those of AQP4 (Figure 1E,G). 

### 3.2. HuNoV Infection Promotes AQP1 Expression

**Figure 2:**
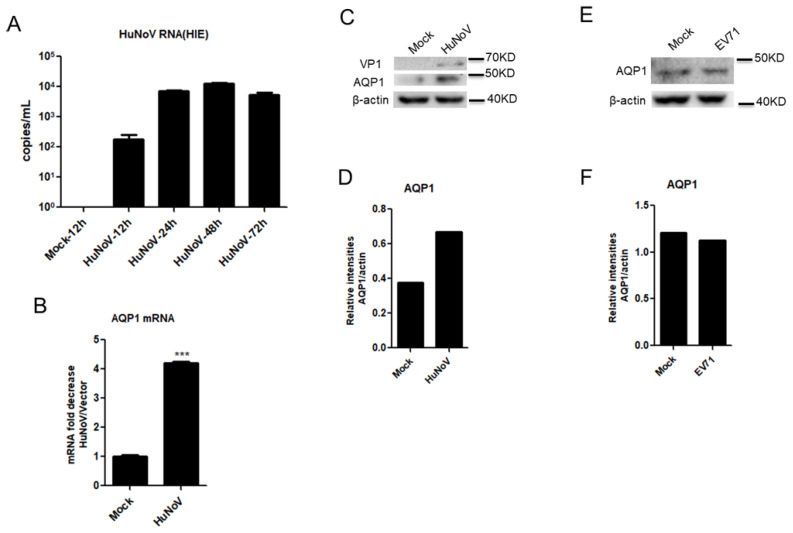
HuNoV infection promotes AQP1 expression. (**A**). HuNoV replicated in HIEs. HIEs were infected with HuNoV at a genome copy of 3.5 × 10^7^. At the indicated time, the copies of HuNoV genome were evaluated by real-time quantitative PCR. (**B**–**D**). HuNoV induced the production of AQP1 at the mRNA and protein levels. HIEs were infected with HuNoV at a genome copy of 3.5 × 10^7^. At 24 h post-infection, the expressions of AQP1 and VP1 were detected by WB (**C**) and quantified using Image J (**D**). The expression of AQP1 was evaluated by relative real-time quantitative PCR (**B**). (**E**,**F**). The enterovirus EV71 did not change the expression of AQP1. Caco2 cells were infected with EV71 at an MOI of 0.05 for 24 h, and the expression of AQP1 was subsequently detected by WB (**E**) and quantified using Image J (**F**). For graphs, data shown are mean ± S.D. of three independent experiments with each condition performed in triplicate. For images, one representative experiment out of three is shown. *** *p* < 0.001.

Having demonstrated that AQP1 expression was upregulated following transfection with complete HuNoV cDNA in Caco2 cells, further experiments were carried out in the context of virus infection. Previous studies showed that HuNoV was able to infect enteric stem cells-derived human intestinal enteroids (HIEs) [16]. We constructed a similar HIE culture system as described previously [30,31,38] to assess the effect of HuNoV on AQP1 expression. Our results showed that progeny viruses produced by our full-length HuNoV cDNA clone productively infected the HIE system (Figure 2A). By using the HIE system, we found that HuNoV induced the expression of AQP1 at both the mRNA and protein levels in the context of virus infection (Figure 2B–D). In contrast, the expression of AQP1 was not affected by the enterovirus EV71, although its infection can cause diarrhea (Figure 2E,F). These results together indicated that HuNoV infection promotes the expression of AQP1.

### 3.3. HuNoV VP1 Promotes the Expression of AQP1

**Figure 3:**
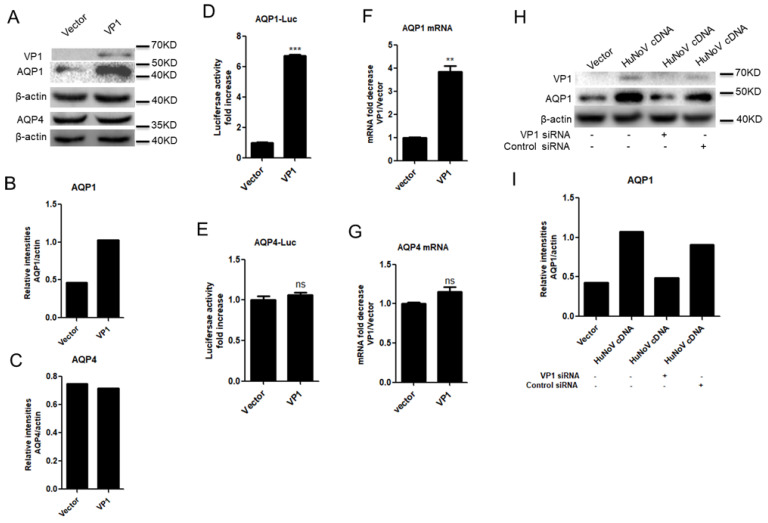
HuNoV VP1 promotes the expression of AQP1. (**A**–**C**). VP1 promoted the production of AQP1. (**D**,**E**). VP1 promoted the activation of AQP1 promoter. Caco2 cells were co-transfected with empty vector or VP1-expressing plasmid and AQP1-Luc or AQP4-Luc for 24 h. Cells were lysed and detected for the activity of Firefly luciferase. (**F**,**G**). VP1 promoted the transcription of AQP1. Caco2 cells were transfected with empty vector or VP1-expressing plasmid for 24 h (**A**,**F**,**G**). The protein levels of AQP1 and VP1 were measured by WB (**A**) and quantified using Image J (**B**,**C**). The transcription of AQP1 and AQP4 was evaluated by relative real-time quantitative PCR (**F**,**G**). AQP1 and AQP4 mRNA copies were normalized using GAPDH and expressed as fold increase in the value for the empty vector. (**H**,**I**). VP1 knockdown impaired HuNoV-induced AQP1 expression. Caco2 cells were transfected with VP1 specific siRNA for 4 h followed by transfection with full length HuNoV cDNA-expressing plasmid for another 24 h. The knockdown effect of VP1 siRNA and AQP1 expression were detected by WB (**H**). The relative intensities of AQP1 blots were quantified (**I**). For graphs, the data shown are mean ± S.D. of three independent experiments with each condition performed in triplicate. For images, one representative experiment out of three is shown. ** *p* < 0.01, *** *p* < 0.001, ns, not significantly.

The genome of HuNoV is organized into three open reading frames (ORFs). ORF2 encodes the major capsid protein VP1, which can form virus-like particles (VLPs) [39]. Considering the important roles of VP1, we first assessed whether VP1 affects AQP1 expression. Caco2 cells were transfected with VP1-expressing plasmid for 24 h. The results indicated that HuNoV VP1 promoted the production of AQP1 in Caco2 cells (Figure 3A–C). HuNoV VP1 also promoted the expression of AQP1 but not AQP4 at both the promoter (Figure 3D,E) and mRNA (Figure 3F,G) levels. The induction of AQP1 expression by HuNoV was blocked when VP1 was knocked down by a VP1-specific siRNA (Figure 3H,I), further indicating the important role of VP1 in HuNoV-mediated AQP1 expression. These results collectively indicated that HuNoV VP1 promotes the expression of AQP1.

### 3.4. HuNoV Promotes AQP1 Expression through NF-κB Signaling Pathway

**Figure 4:**
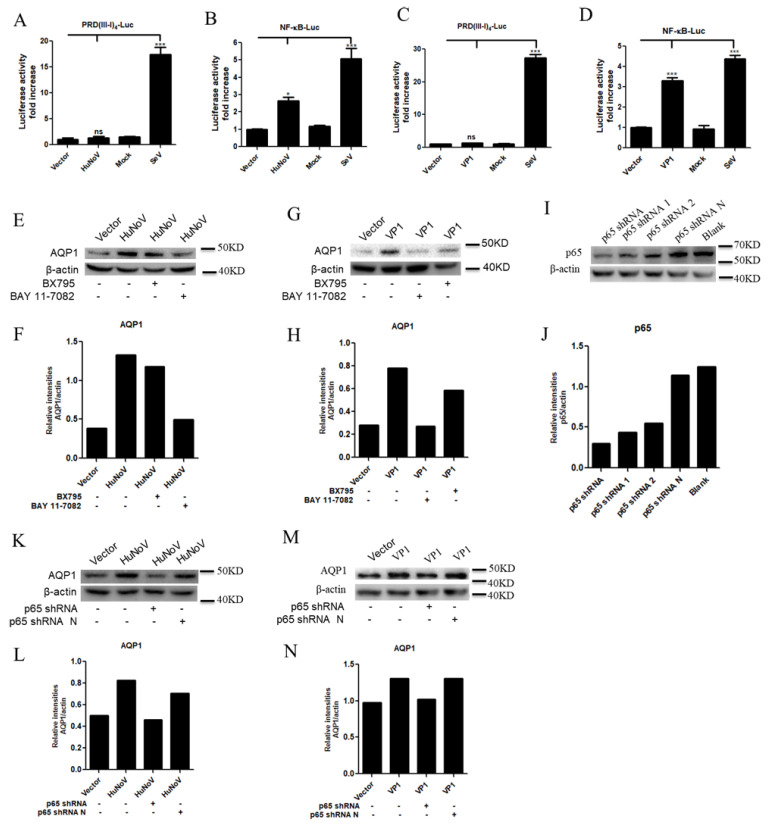
HuNoV promotes AQP1 expression through NF-κB signaling pathway. (**A**,**B**). HuNoV promoted the activation of NF-κB-responsive promoter. (**C**,**D**). VP1 promoted the activation of NF-κB-responsive promoter. Caco2 cells were transfected with PRD(III-I)_4_-Luc or NF-κB-Luc for 4 h followed by stimulation with or without 100 HAU mL^−1^ SeV, or co-transfected with empty vector, full-length HuNoV cDNA (**A**,**B**) or VP1 (**C**,**D**)-expressing plasmid and PRD(III-I)_4_-Luc or NF-κB-Luc. (**E**–**H**). HuNoV and VP1 promoted the expression of AQP1 through NF-κB signaling pathway. Caco2 cells were transfected with empty vector or full-length HuNoV cDNA (**E**) or VP1 (**G**)-expressing plasmid. The inhibitor BX795 or BAY 11-7082 were added at 4 h post-transfection. At 24 h post-transfection, the expression of AQP1 was detected by WB (**E**,**G**) and quantified using Image J (**F**,**H**). (**I**–**N**). p65 knockdown blocked HuNoV or VP1-induced AQP1 expression. Caco2 cells were transfected with or without different p65 specific shRNA or control shRNA (p65 shRNA N) (**I**,**J**). The knockdown effect of p65 shRNA was detected by WB (**I**) and quantified using Image J (**J**). p65 specific shRNA or p65 shRNA N was chosen to be transfected into Caco2 cells. At 24 h post-transfection, cells were transfected with empty vector or full-length HuNoV cDNA (**K**) or VP1 (**M**)-expressing plasmid for 24 h. The expression of AQP1 was detected by WB (**K**,**M**) and quantified using Image J (**L**,**N**). Data shown are mean ± S.D. of three independent experiments with each condition performed in triplicate. One representative experiment out of three is shown. * *p* < 0.05, *** *p* < 0.001, ns, not significantly. N, negative control.

HuNoV is one of the most frequent causes of epidemic gastroenteritis [39,40]. Furthermore, MNV activates inflammatory responses in mice [41,42,43]. Due to the critical role of the NF-κB signaling pathway in inducing the inflammatory responses, we therefore investigated whether the abnormal expression of AQP1 mediated by HuNoV is associated with the NF-κB signaling pathway. Caco2 cells were co-transfected with IRF3 (Interferon regulatory Factor 3, IRF3) or NF-κB-responsive reporter plasmid PRD(III-I)_4_-Luc or NF-κB-Luc and plasmid expressing full-length HuNoV cDNA or VP1 for 24 h. Our results showed that both HuNoV (Figure 4A,B) and VP1 (Figure 4C,D) induced the activation of NF-κB but not IRF3-responsive promoter, while the two promoters could be activated by SeV. By using the inhibitor BX795 of the IRF-3 signaling pathway or the inhibitor BAY11-7089 of the NF-κB signaling pathway, we found that the expression of AQP1 induced by HuNoV or VP1 was through the NF-κB signaling pathway (Figure 4E–H). Furthermore, the induction of AQP1 expression by HuNoV or VP1 was blocked (Figure 4K–N) when p65, a major component of NF-κB, was knocked down (Figure 4I,J). These results together indicated that HuNoV promotes AQP1 expression through the NF-κB signaling pathway.

### 3.5. HuNoV Induces the Expression, Phosphorylation and Nuclear Translocation of p65

**Figure 5:**
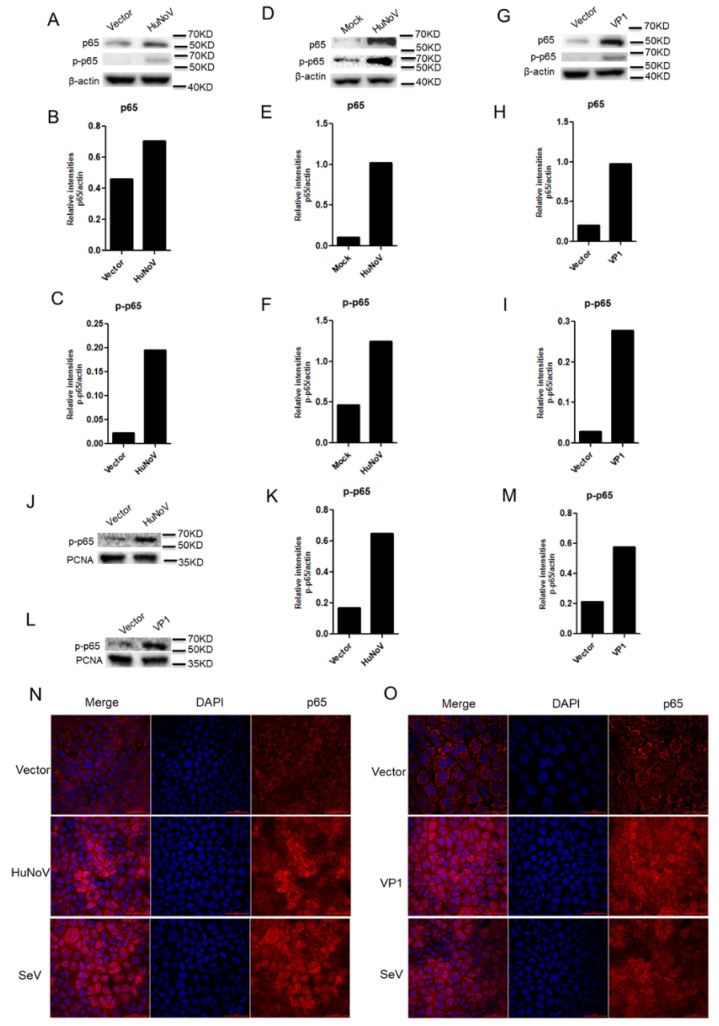
HuNoV induces the expression, phosphorylation and nuclear translocation of p65. (**A**–**F**). HuNoV induced the expression and phosphorylation of p65. (**G**–**I**). VP1 induced the expression and phosphorylation of p65. Caco2 cells were transfected with empty vector or full-length HuNoV cDNA (**A**) or VP1 (**G**)-expressing plasmid for 24 h. HIEs (**D**) were infected with HuNoV at a genome copy of 3.5 × 10^7^ for 24 h. The expressions of p65 and phosphorylated p65 were detected by WB (**A**,**D**,**G**) and quantified using Image J (**B**,**C**,**E**,**F**,**H**,**I**). (**J**,**K**,**N**). HuNoV induced the nuclear translocation of p65. (**L**,**M**,**O**). VP1 induced the nuclear translocation of p65. Caco2 cells were transfected with empty vector or full-length HuNoV cDNA (**J**) or VP1 (**L**)-expressing plasmid for 24 h, followed by isolation of nuclear proteins from cells. The phosphorylated p65 in nuclei was detected by WB (**J**,**L**) and quantified using Image J (**K**,**M**). Caco2 cells in 35 mm dishes were transfected with full-length HuNoV cDNA (**N**) or VP1 (**O**)-expressing plasmid, or directly stimulated with 100 HAU mL^−1^ SeV for 24 h. Cells were stained with a rabbit anti-p65 Ab, which was followed by incubation with Alexa Fluor 647-conjugated goat anti-rabbit (red) as the secondary Ab. Cell nuclei (blue) were stained with Hoechst 33258. The phosphorylated p65 in nuclei was observed by IFA (**N**,**O**). The images were obtained by fluorescence microscopy using 60× objective. One representative experiment out of three is shown. The scale bar indicates 50 μm.

The p65 subunit is a major component of NF-κB complexes, which is responsible for trans-activation of gene expression [44]. To further address the underlying mechanism of HuNoV interaction with the NF-κB signaling pathway, Caco2 cells were transfected with the full-length HuNoV cDNA for 24 h to assess the interaction of HuNoV with p65. The results showed that the expression and phosphorylation of p65 were induced after cells were transfected with the full-length HuNoV cDNA (Figure 5A–C), which were further confirmed in the context of HuNoV infection (Figure 5D–F). Likewise, HuNoV VP1 also promoted the expression and phosphorylation of p65 (Figure 5G–I). In most cell types, NF-κB dimers are usually inactivated and kept in the cytoplasm. Only when NF-κB was activated and translocated into nucleus, the transcription of genes could be mediated by NF-κB [45]. We next examined whether HuNoV induces the nuclear translocation of p65. Caco2 cells were transfected with the full-length HuNoV cDNA or VP1 for 24 h. Cells were collected to isolate nuclear proteins for WB assay (Figure 5J–M) or used to observe the location of p65 by IFA (Figure 4N,O). The results showed that phosphorylated p65 in the nucleus was obviously increased in HuNoV-transfected cells (Figure 5J,K,N), VP1-transfected cells (Figure 5L,M,O), or SeV-infected cells (Figure 5N,O). These results collectively indicated that HuNoV and VP1 activates the NF-κB signaling pathway by inducing the expression, phosphorylation and nuclear translocation of p65.

### 3.6. HuNoV Increases the Permeability of Intestinal Epithelial Barrier

**Figure 6:**
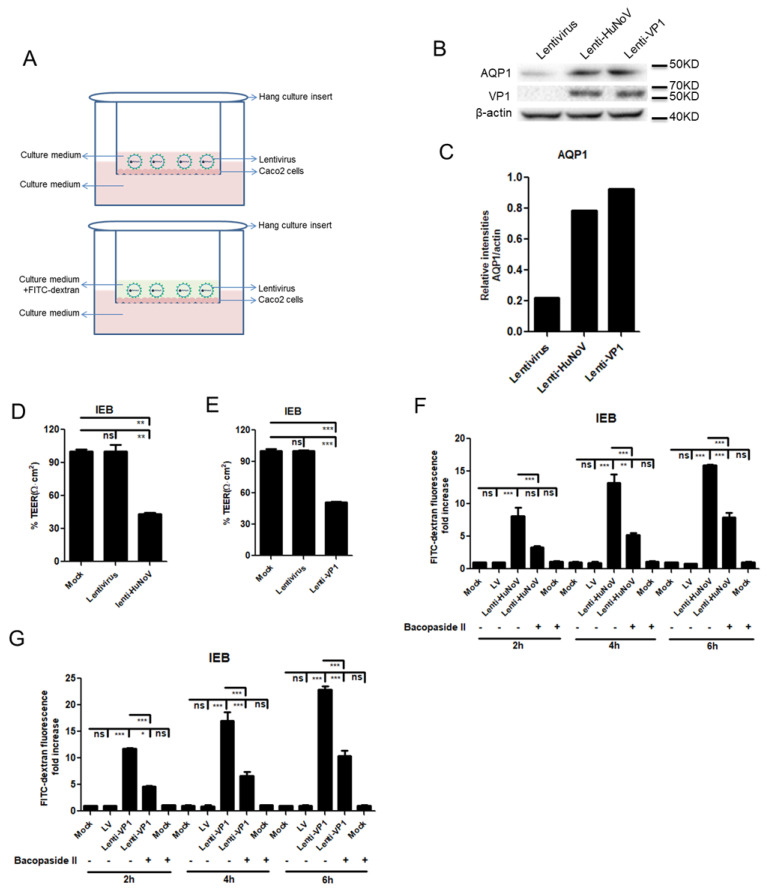
HuNoV increases the permeability of intestinal epithelial barrier. (**A**). The model of IEB. (**B**,**C**). Lenti-HuNoV or Lenti-VP1 transduction induced the production of AQP1. (**D**,**E**). HuNoV and VP1 significantly decreased the value of TEER. (**F**,**G**). HuNoV and VP1 promoted penetration of small molecular FITC–dextran. IEBs were transduced with empty lentivirus, lenti-HuNoV or lenti-VP1 at an MOI of 1 for 3 d. Cells were lysed, and the expression of AQP1 was detected by WB (**B**). The relative intensities of AQP1 blots were quantified (**C**). The value of TEER was measured by voltmeter (**D**,**E**). The IEB were pretreated with or without Bacopaside II for 1 h before FITC-conjugated dextran (1 mg/mL) was added to the upper chamber of Transwells. At the indicated time, 100 μL of sample in the lower chamber was collected and quantified using VARIOSKAN FLASH (Thermo Scientific) (**F**,**G**). For graphs, data shown are the mean ± S.D. of three independent experiments with each condition performed in triplicate. For images, one representative experiment out of three is shown. * *p* < 0.05, ** *p* < 0.01, *** *p* < 0.001, ns, not significantly. LV, empty lentivirus.

To address the physiological relevance of HuNoV-induced AQP1 expression, we constructed a human intestinal epithelial barrier (IEB) to detect the effect of HuNoV on its transepithelial electrical resistance (TEER). TEER value is a widely accepted quantitative indicator of the integrity of IEB [46,47,48,49]. Considering its polarized characteristic, Caco2 cells were commonly chosen to construct the human IEB system [48,49,50,51,52]. The model of IEB using Caco2 cells in this study is shown in Figure 6A. Due to very low transfection efficiency in IEB, we constructed HuNoV and VP1-expressing lentivirus, respectively, for transduction. Firstly, the expression of AQP1 was detected after transduction with a recombinant lentivirus carrying HuNoV (named lenti-HuNoV) or VP1 (named lenti-VP1). The induction of AQP1 expression was observed after transduction with lenti-HuNoV or lenti-VP1 (Figure 6B,C). Moreover, following transduction with lenti-HuNoV (Figure 6D) or lenti-VP1 (Figure 6E), the TEER value of IEB significantly decreased compared to that with the empty lentivirus, indicating that the integrity of IEB was likely abrogated by HuNoV and VP1. We next assessed whether HuNoV or VP1 affected the permeability of IEB using small molecule FITC-conjugating dextran (FITC-dextran). The results showed that HuNoV (Figure 6F) and VP1 (Figure 6G) enhanced the penetration of FITC-dextran through IEB, which was significantly blocked when the AQP1 channel was inhibited by Bacopaside II. These results together indicated that HuNoV increases the permeability of IEB and that HuNoV-mediated enhancement of small molecule permeability is associated with the AQP1 channel.

## 4. Discussion

HuNoV represents one-fifth of episodes of acute gastroenteritis across all ages, and it results in more than USD 4 billion in direct medical costs and more than USD 60 billion in indirect societal costs [10]. Although the World Health Organization in 2016 stated that it should be an absolute priority to develop a HuNoV vaccine, unfortunately, there is currently no licensed HuNoV vaccine and specific antiviral available. The major barrier to the basic research and prevention of HuNoV is the lack of a robust and reproducible in vitro cultivation system. It remains elusive as to how HuNoV infection causes diarrhea. 

In this study, we found that HuNoV transfection induced the expression of AQP1, which was further confirmed in the context of virus infection. Of interest, although HuNoV infection induced the expression of AQP4 in HIEs (data not showed), AQP4 expression was not affected in Caco2 cells transfected with full-length HuNoV cDNA. We confirmed that a full-length HuNoV cDNA clone produced progeny viruses containing an intact RNA genome with the size similar to that of HuNoV, although the progeny viruses were unable to infect Caco2 cells (Supplementary Materials Figure S1A–E). Given that HuNoV transfection features a lack of the viral entry process compared with HuNoV infection, HuNoV-mediated AQP4 expression is likely associated with cellular responses induced by viral entry. 

We next identified HuNoV VP1 as the key viral component in promoting AQP1 expression in Caco2 cells. AQPs play a key role in regulating the dynamic balance of water [25,26]. When the expression of AQPs on intestinal epithelial cells decreases, the secretion of the intestine exceeds its absorption, which will inevitably lead to diarrhea. Vice versa, when the absorption exceeds the secretion, it could result in the water in plasma quickly entering the intestinal lumen in order to maintain the balance of osmotic pressure. An influx of liquid in a short time would also cause diarrhea. Therefore, AQP upregulation or downregulation could both lead to diarrhea. It is reasonable to conclude that the upregulation of AQP1 induced by HuNoV may at least in part contribute to HuNoV-induced diarrhea.

We further addressed the potential molecular mechanism underlying HuNoV-induced AQP1 expression, showing that HuNoV and VP1 mediated the expression of AQP1 through the NF-κB signaling pathway. In agreement with our findings, a previous study by others showed that the upregulation of AQP3 expression is associated with the NF-κB signaling pathway [36]. HuNoV is a major cause of severe gastroenteritis worldwide [5,53]. If HuNoV activated the NF-κB signaling pathway, this could inevitably result in the expression of inflammatory cytokines, contributing to the occurrence and development of HuNoV-induced gastroenteritis. Given that diarrhea is a self-limited disease and generally recovers in a week, such NF-κB activation may contribute to the clearance of HuNoV by the host. P65 is a key component of NF-κB, playing a vital role in the activation of the NF-κB signaling pathway. It is known that many viruses affect the NF-κB signaling pathway by acting on p65 [54,55,56,57,58]. In this study, we demonstrated that HuNoV and VP1 promoted the expression, phosphorylation and nuclear translocation of p65, revealing that HuNoV activates the NF-κB signaling pathway by acting on p65.

Following demonstration that HuNoV induced the expression of AQP1, we assessed whether AQP1 abnormal expression is associated with diarrhea. We constructed a model to mimic the human intestinal epithelial barrier (IEB) using Caco2 cells. We found that both HuNoV and VP1 could significantly induce the decline of the trans-epithelial electrical resistance (TEER) value of IEB and promote the penetration of FITC–dextran through IEB, which was significantly blocked when the AQP1 channel was inhibited. These findings indicated that the permeability of IEB enhanced by HuNoV and VP1 is associated with the AQP1 channel, revealing a possible mechanism underlying HuNoV-caused diarrhea.

## 5. Conclusions

In conclusion, we found that HuNoV induces the production of AQP1 via activating the NF-κB signaling pathway. Moreover, the permeability of IEB enhanced by HuNoV and VP1 is associated with the AQP1 channel. The findings in this study would be important not only for understanding the potential mechanism underlying how HuNoV causes diarrhea but also for the development of drug candidates for the treatment of such diarrhea.

## Data Availability

Not applicable.

## Supplementary Materials

The following supporting information can be downloaded at: https://www.mdpi.com/article/10.3390/v14040842/s1, Figure S1: HuNoV complete cDNA produces progeny viruses containing intact RNA genome; Table S1: Primers used in this study.
