# Supplementary material for: Human Norovirus Induces Aquaporin 1 Production by Activating NF-κB Signaling Pathway
Source: Viruses. 2022 Apr 18;14(4):842. doi: 10.3390/v14040842 (PMC9028284; doi:10.3390/v14040842)
Supplement: Supplementary file 1 [file viruses-14-00842-s001.zip › viruses-1667805-supplementary.pdf]

Table S1. Primers used in this study

For plasmid construction	
Primer Name	Sequence [a]
VP1-C3F-F	5'- GTTTAAACTTAAGCTATGAAGATGGCGTCGAATGA -3’
VP1-C3F-R	5'-CTGGACTAGTGGAT**CCTACTTATCGTCGTCATCCTTG TAATCCTTATCGTCGTCATCCTTGTAATCCTTATCGTC GTCATCCTTGTAATC**TAATGCACGTCTACGCC -3’
AQP1-Luc-F	TCTCTATCGATAGGTACCGTCTCTTTCACTTCCAC
AQP1-Luc-R	TACCGGAATGCCAAGCTTGCTGGCAGGGGGCTTGGC
AQP4-Luc-F	TCTCTATCGATAGGTACCCATTGTTCTAGATGTTG
AQP4-Luc-R	TACCGGAATGCCAAGCTTGCCTTCCCCAGCCAGAG
For real time PCR	
AQP1-F	5'-CACGACCCTCTTTGTCTTCA-3'
AQP1-R	5'-ACGCACTGGGCGATGATGTA-3'
AQP4-F	5'-AGTGACAGACCCACAGCAAG-3'
AQP4-R	5'-CAAAGCAAAGGGAGATGAGA-3'
GAPDH-F	5'-GGGAAGCTCACTGGCATGG-3'
GAPDH-R	5'-TTACTCCTTGGAGGCCATGT-3'

[a] Flag sequences are highlighted in bold.

F, forward. R, reverse.

Figure S1

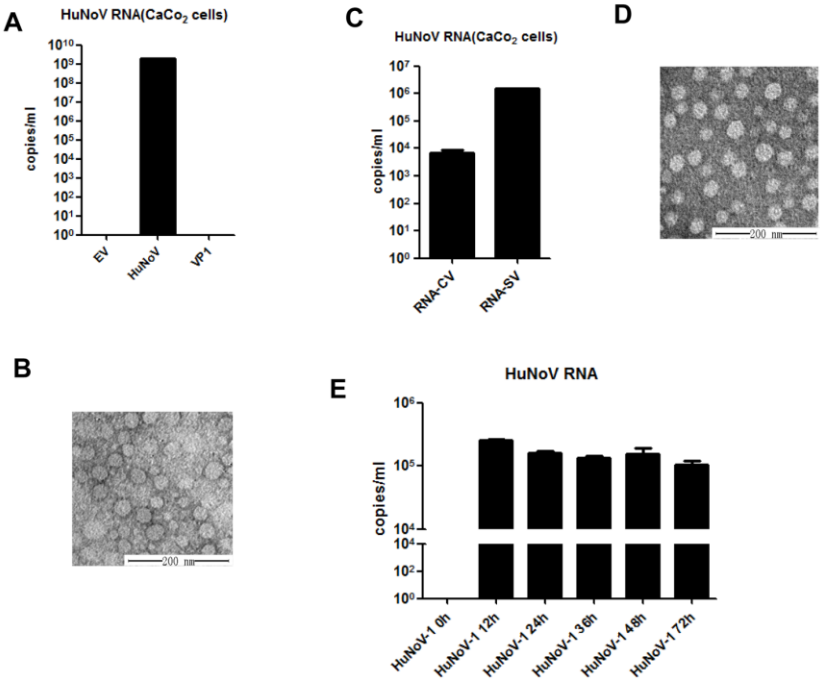

Figure S1. HuNoV complete cDNA produces progeny virus containing intact RNA genome. A. HuNoV replication. B. Production of HuNoV progeny virus. Caco2 cells in 6-well plates were transfected with 3 μg HuNoV or VP1-expressing plasmid or empty vector for 3 d. Cells were harvested and total RNA was extracted. The copies of HuNoV genome were evaluated by real-time quantitative PCR (A). HuNoV in the cell supernatant was observed under transmission electron microscope (B). C. Functionality of HuNoV RNA genome. D. Production of HuNoV progeny virus. The RNA genome of progeny virus was extracted and subsequently transfected into Caco2 cells for 3 d. The viral RNA in supernatant (SV) and cells (CV) were extracted and quantified by real-time quantitative PCR (C). Virus in the cell supernatant was observed under transmission electron microscope (D). E. HuNoV progeny virus does not infect Caco2 cells. Caco2 cells were infected with HuNoV progeny virus at a genome copy of 3.5×10^{7}. Cells were harvested at the indicated time points following

infection. RNAs were extracted and quantified by real-time quantitative PCR. For graphs, data shown are mean ± S.D. of three independent experiments with each condition performed in triplicate. For images, one representative experiment out of three is shown. EV, empty vector. The scale bars indicated 200 nm.
